# Evaluating a new inpatient program for adolescent AN: impacts on admission rates and patient outcomes

**DOI:** 10.1186/2050-2974-3-S1-O9

**Published:** 2015-11-23

**Authors:** Eva Vall, Mandy Yiu, Michael Batterham, Tracey Wade

**Affiliations:** 1 Flinders University, Australia; 2 Flinders Medical Centre, Australia

## 

Anorexia nervosa (AN) is particularly dangerous for adolescent sufferers, as the potential for long term damage is heightened during this critical developmental period. Hospitalisation is necessary for many adolescents with AN, and in Australia, around 90% of such admissions are to public hospitals. Given the high costs associated with this treatment modality, the economic impact on public health systems is substantial. In 2013, a new adolescent eating disorder program was introduced in a large public paediatric unit. Informed by the Maudsley Model, the program had the dual aim of improving patient outcomes and managing service demand. We evaluated the program's success in terms of its impact on admission rates and duration, and on patient outcomes. Despite an increased demand for inpatient services since the new program commenced, it has achieved significant reductions in occupied bed days without compromising patient outcomes. Weight gain and decreases in eating psychopathology were maintained at 3-month follow-up. This presentation will detail the results of the evaluation, and discuss its implications for other services facing similar challenges. We will also discuss the implications of this research for informing improvements in the provision of effective inpatient treatment for adolescent AN.

